# PHARMACOLOGIC AMPK ACTIVATION IMPROVES HEALTHSPAN AND LIFESPAN IN NORMAL AND PREMATURE AGING MODELS

**DOI:** 10.1093/geroni/igad104.3524

**Published:** 2023-12-21

**Authors:** Ozlem Altintas Akin, Sarah Mitchell, Shana Sturla, Michael MacArthur

**Affiliations:** ETH Zurich, Zurich, Zurich, Switzerland; Princeton University, Princeton, New Jersey, United States; ETH Zurich, Zurich, Zurich, Switzerland; Princeton University, Princeton, New Jersey, United States

## Abstract

Cockayne Syndrome (CS) is a rare autosomal recessive childhood disease, caused by mutations in CSA (ERCC8) or CSB (ERCC6) genes leading to defective DNA damage repair. CS characteristics include UV sensitivity and premature aging. Deficiencies in CSA or CSB may lead to impaired AMP-activated protein kinase (AMPK) activation. Notably, AMPK-activating interventions have shown promise in preclinical CS mouse models. We tested whether MK8722, an AMPK activator, could mitigate CS phenotypes in a C. elegans model. Our preliminary data show that MK-8722 increases lifespan in wild-type and CS worm model. We also tested the effect of MK-8722 on brood size as a fitness parameter and showed that it does not change the total brood size. We also showed that treating aged mice with MK-8722 improves multiple measures of healthspan including body composition, grip strength and age-associated inflammation. These data suggest that AMPK activation may be a promising therapeutic avenue for both normal and CS-associated aging phenotypes

